# Knowledge, Attitudes, Practices and Some Characteristic Features of People Recovered from COVID-19 in Turkey

**DOI:** 10.3390/medicina57050431

**Published:** 2021-04-30

**Authors:** Seda Yakut, Burcu Karagülle, Tuğçe Atçalı, Yasin Öztürk, Mehmet Nuri Açık, Burhan Çetinkaya

**Affiliations:** 1 Department of Histology and Embryology, Faculty of Veterinary Medicine, University of Bingol, 12000 Bingol, Turkey; syakut@bingol.edu.tr; 2 Department of Microbiology, Faculty of Veterinary Medicine, University of Firat, 23119 Elazig, Turkey; bkaragulle@firat.edu.tr (B.K.); bcetinkaya@firat.edu.tr (B.Ç.); 3 Department of Physiology, Faculty of Veterinary Medicine, University of Bingol, 12000 Bingol, Turkey; tatcali@bingol.edu.tr; 4 Department of Pharmacology, Faculty of Veterinary Medicine, University of Bingol, 12000 Bingol, Turkey; yasinozturk@bingol.edu.tr; 5 Department of Microbiology, Faculty of Veterinary Medicine, University of Bingol, 12000 Bingol, Turkey

**Keywords:** COVID-19, questionnaire, knowledge, attitude, practice

## Abstract

*Background and objectives:* The whole world is spending an extraordinary effort by implementing various measures to control and prevent the COVID-19 pandemic. The effectiveness of the preventive measures is greatly influenced by the public’s knowledge, attitudes, and practices (KAP) towards the disease. In this study, KAP values and some characteristic features of people recovered from COVID-19 were determined by conducting a questionnaire survey. *Materials and Methods:* The questionnaire survey was conducted between 1 and 10 January 2021 on people who recovered from COVID-19 in a total of 150 different locations in Turkey. The questionnaire consisted of 46 questions: 14 for determining demographic and some characteristic features of the participants, and 32 for determining their knowledge, attitudes, and practices. The data obtained were evaluated using descriptive statistics, chi-squared tests, *t*-tests, and one-way analysis of variance (ANOVA). *Results*: It was determined that 63% of the participants had at least one chronic illness, 3.9% suffered from the disease twice, and 45.2% changed their smoking habits. The average knowledge score of the participants about COVID-19 was calculated as 10.25 (SD = 2.37; range 0–15). The participants were found to have a high level of knowledge about the symptoms and prevention methods in general, and positive changes in post-illness attitudes and behaviors. However, there was a great instability regarding the drugs and vaccines used in the treatment of COVID-19. *Conclusions:* This was the first study carried out in Turkey to determine knowledge, attitudes, practices, and some characteristic features of people who recovered from COVID-19. It was suggested that health authorities in the country need to develop more effective strategies and policies to find out permanent solutions in order to control and prevent the COVID-19 pandemic by taking into account the concerns of the public, particularly with regards to the drugs used in the treatment and vaccination.

## 1. Introduction

Coronavirus disease 2019 (COVID-19), an acute respiratory disease caused by the SARS CoV-2 virus, was first reported in Wuhan, China, in late 2019 [1]. The first COVID-19 case was officially reported on March 9, while the first death due to the disease on 17 March 2020 in Turkey [2]. Then, the disease spread rapidly across the country owing to human movements. Following the emergence of the first case, the Turkish government started to take many strict measures such as closing schools, shopping centers, and other workplaces, as well as imposing flexible working hours, travel restrictions, and curfews to prevent the transmission and spread of the disease. In addition to announcements and posters, continuous briefing throughout social media were put into place in order to inform the public about this disease and raise awareness for taking effective precautions.

Implementation of preventive measures and infection control procedures is extremely important to reduce the spread of this disease. The effectiveness of the COVID-19 preventive measures is greatly influenced by knowledge, attitudes, and practices (KAP) of people towards the disease. The huge amount of misinformation/disinformation shared on social media that overshadow people’s understanding of COVID-19 cause great confusion among the public. KAP data for COVID-19 plays an important role in determining whether a society is ready to accept measures concerning behavioral changes suggested by health authorities [3]. KAP studies provide basic information on determining the type of intervention that may be needed to change myths about the virus. Evaluating the KAP on COVID-19 in the society will help create better planning and strategies for preventing insufficient and/or misinformation about the disease and for developing health promotion programs. Many surveys have been conducted all over the world to investigate the knowledge, attitudes, and practices of people from different socio-cultural strata and professions against COVID-19 [4,5,6]. However, the number of studies establishing KAP data of people who suffered from the disease is limited [7]. Therefore, there is a paucity of information about the possible changes on KAP data of people who recovered from COVID-19 when compared with those without the disease. The present study was carried out to determine the knowledge, attitudes, and practices of people who recovered from COVID-19 in Turkey. In addition, the views of the participants about some characteristic features were measured. The results of this study are expected to provide useful information to policymakers for developing better control and prevention measures against COVID-19 outbreak.

## 2. Materials and Methods

### 2.1. Ethical Approval

A formal permission was obtained from the Ministry of Health to carry out this questionnaire survey. The study was also ethically approved by the Scientific Research and Publication Ethics Committee of Bingol University with the protocol number 30/12/2020-E.23861.

### 2.2. Survey Region and Sample Size

Turkey is a country comprising seven geographic regions and 81 provinces with a total of 83 million population in an area of 780,000 km^2^. The total number of COVID-19 cases in Turkey as of 31 December 2020 was approximately 2,200,000. According to the data obtained from the Ministry of Health, the vast majority of these cases occurred in the western provinces of the country where the population density is higher. The questionnaire survey was applied in a total of 150 different points located in 55 provinces and 95 towns that were representative of all the geographical regions in the country. While conducting the survey, we paid particular attention to the participation of people residing in provinces with higher population density and COVID-19 cases. The sample size was calculated by using the Raosoft sample size calculation method based on 99% confidence interval, 5% error rate, and 50% response distribution [8]. According to this method, we calculated the sample size as 664 people among the total number of 2,200,000 COVID-19 cases in Turkey.

### 2.3. Survey Method

The questionnaire survey was conducted between 1 and 10 January 2021 on people who recovered from COVID-19 in Turkey. Before conducting the survey, we carried out a pilot study on randomly selected 30 participants who recovered from COVID-19 in Bingol and Elazig provinces in order to test the clarity, reliability, and validity of the questionnaire. Then, the questionnaire was revised and finalized in line with the recommendations, and data were collected from the study population. The questionnaire, consisting of five parts and a total of 46 questions, was established by considering previous similar studies [6,9]. The survey was conducted by using online data collection method in order to avoid close contact due to the fact that COVID-19 is very contagious. The questionnaire was prepared using Google Forms data collection method. The link of the questionnaire was shared on WhatsApp and Facebook websites, which are the most popular social media communication tools, in order to reach the participants. In addition, the questionnaire form was loaded on other social media sites such as Instagram and Twitter. Before the questionnaire was filled out, a brief information was provided to explain the aim and significance of the survey and to encourage the participants to response the questionnaire. In addition, it was stated in the information section that only one person from each family should participate in the questionnaire in order to obtain more accurate and representative results. The participants were first asked whether they had COVID-19, and those who answered no (*n* = 118) to this question were prevented from completing the questionnaire. On the other hand, the participants who answered yes (*n* = 664) to this question were allowed to access the questionnaire. Hence, the proportion of those who had the disease was calculated as 85% (664/782) among the total of number of participants entering the questionnaire system. In the questionnaire, a total of 46 questions were asked to the participants, including 6 questions for demographic information, 8 for determining some characteristics of the participants, 15 for measuring their knowledge about COVID-19, 9 for measuring their attitudes about the disease, and 8 questions for measuring their practices regarding the disease. While the first two parts (demographic and some characteristic features of the participants) of the questionnaire included close-ended questions, the questions measuring knowledge, attitudes, and practices of the participants were presented with the options of true, false, and not sure.

### 2.4. Statistical Analysis

SPSS (Statistical Package for Social Sciences for Windows, Release ver.26.0) package program was used for the statistical analysis of the data. The diverging stacked bar charts for three-point Likert scales were generated by Tableau 8.2 (Tableau Software, Washington, DC, USA). A total of 15 items were used to measure knowledge about COVID-19. The participants responded to these items by choosing either “true”, “false”, or “not sure”, and a correct response to an item was assigned as 1 point, while an incorrect/not sure response was assigned as 0 points. The maximum total score ranged from 0 to 15. As the score increased in this range, knowledge about COVID-19 increased as well. The scores (yes = 1 point, no = 2 points, not sure = 3 points) obtained from the questions regarding the attitudes and practices of the participants towards COVID-19 were collected and the section scores were calculated. The Cronbach’s alpha reliability coefficient was calculated as 0.747. Knowledge scores were categorized according to Bloom’s cut-off points as 80.0–100.0% (high level, 12–15 points), 60.0–79.0% (medium level, 9–11points), and ≤59.0% (low level, 0–8 points). The knowledge score values demonstrated as mean (x̄) and standard deviation (SD). Descriptive statistics focused on frequencies and percentages that were used for analytical comparisons between characteristic features, knowledge, attitudes, and practices of the participants related to COVID-19. Normality of data was assessed with skewness and kurtosis, whereas Levene’s test was used to validate variance homogeneity. Independent samples *t*-tests or one-way analysis of variance (ANOVA) followed by Tukey’s post hoc were employed for the data that fits parametric assumptions to determine the differences between groups for selected demographic variables. The chi-squared independence test was used to detect whether there was a relationship between the approaches of the participants to vaccination with COVID-19 vaccines and demographic parameters. The statistical significance level was set at *p* < 0.05 for all analyses.

## 3. Results

### 3.1. Demographic Data

Analysis of demographic parameters showed that the majority of the participants were female (51.1%) and in the age range of 25–36 years old (36.3%). In addition, most of the participants stated that they were married (64%), had an associates/bachelor’s degree (56.1%), and worked as a civil servant (59%). Other demographic features were presented in Table 1.

### 3.2. Data on Some Characteristic Features of the Participants

It was determined that 30% of the participants in the survey smoked before contracting the disease, but 45.2% of the smokers changed their smoking attitude (quit or reduced smoking) after they overcame the disease. Most of the participants stated that they had a meat-based diet (34.9%), and the number of those with a chronic disease was found to be quite high (63%). High blood pressure (8.1%), allergy (7.7%), anemia (6.6%), and diabetes (6.5%) were the most frequently reported chronic diseases. Interestingly, 3.9% of the participants pointed out that they contracted COVID-19 twice, while 5.6% of them spent the treatment process in the hospital. One of the most striking finding of the survey was that 22.7% of the participants refused taking COVID-19 treatment (Table 2).

### 3.3. Data on Knowledge Levels and Scores of the Participants in Relation with COVID-19

**Figure 1:**
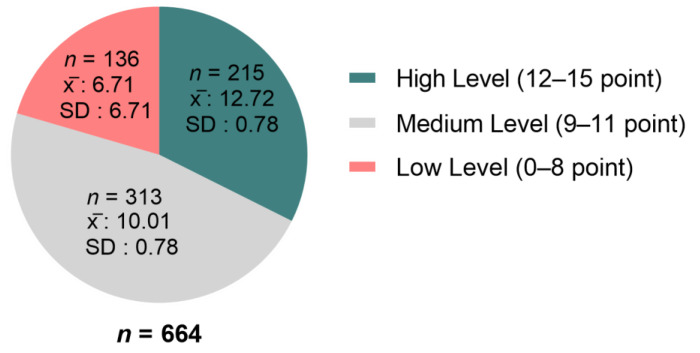
Distribution of knowledge score level of participants. x̄: mean, SD: standard deviation. Knowledge scores were categorized according to Bloom’s cut-off points as 80.0%–100.0% (high level, 12–15 points), 60.0%–79.0% (medium level, 9–11 points), and ≤59.0% (low level, 0–8 points).

**Figure 2:**
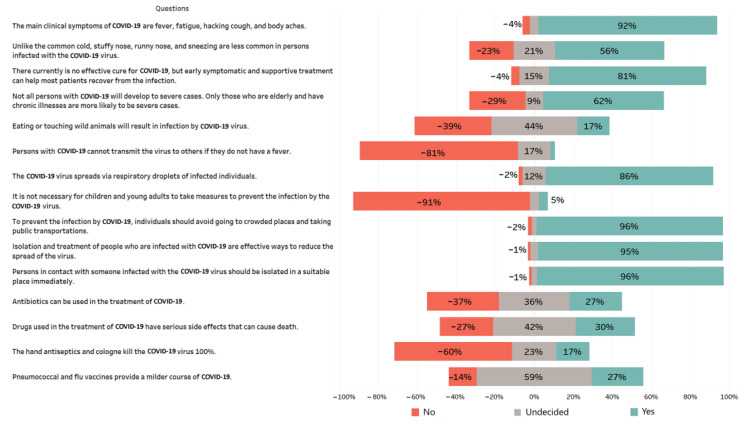
Knowledge levels of the participant on COVID-19 (*n* = 664).

A total of 15 questions were asked to measure the knowledge of the participants about COVID-19, and the average knowledge score was determined as 10.25 (SD = 2.37; in the range of 0–15). While 32.4% (x̄ = 12.72) of the participants had high knowledge score, the percentage for low knowledge score was determined as 20.5% (x̄ = 6.71) (Figure 1). While the overall correct response rate of the questionnaire was 68.35%, the range for all the participants was found to vary between 26.5% and 95.9%. Most of the participants (91.7%) reported that the main clinical symptoms of COVID-19 were fever, fatigue, hacking cough, and body aches. Nearly all of the participants (95.9%) were aware that contact with someone infected with the COVID-19 virus required isolation immediately. The overall results showed that the participants had sufficient information about the symptoms of the disease and were well aware of the protective measures in general. However, they did not have sufficient information about the efficacy of the measures taken against the virus. For instance, the participants gave wrong answers to most of the questions related to information on vaccines and antibiotics. They also did not have correct information whether the virus was transportable by animals (Figure 2).

The analysis results of differences in knowledge scores between gender, age, marital status, education level, place of residence, and occupation groups are presented in Table 3. According to the results, knowledge score varied significantly by the education level (*p* < 0.05). It was determined that the knowledge score belonging to the participants with postgraduate education level was the highest (x̄ = 10.94), whereas it was the lowest for those with high school education level (x̄ = 9.50). Another variable that showed significant difference in knowledge score was place of residence. The knowledge scores of the participants who settled down in provinces, towns, and villages were determined as x̄ = 10.41, x̄ = 10.24, and x̄ = 9.50, respectively. According to the results, there was a positive correlation between the size of living area and the rate of knowledge level. In addition, the knowledge score was observed to vary significantly according to the type of occupation, and civil servants (x̄ = 10.61) were found to be more knowledgeable about COVID-19.

### 3.4. Attitudes of the Participants towards COVID-19

**Figure 3:**
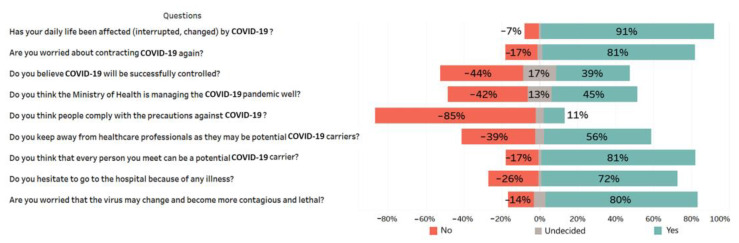
Attitudes of the participants toward COVID-19 (*n* = 664).

Of the participants, 91.4% stated that their daily lives were interrupted by the COVID-19 pandemic, and 80.6% were worried about contracting the disease again. The number of the participants believing that COVID-19 could be successfully controlled was found to be quite low (38.7%). More than half of the participants reported that they avoided from close contact with healthcare workers as they could be potential COVID-19 carriers. Other data concerning the attitudes of the participants towards COVID-19 were presented in Figure 3.

### 3.5. Practices of the Participants towards COVID-19

**Figure 4:**
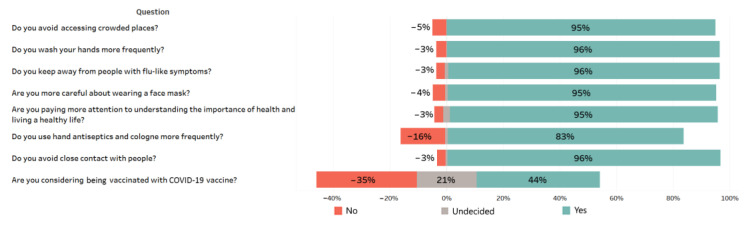
Practices of the participants toward COVID-19 (*n* = 664).

It was observed that there was a high rate of positive change in the practices of the participants regarding COVID-19. A very high proportion of the participants were observed to show great sensibility in practices such as social distancing, using masks, hand washing, and staying away from the crowd, and they were determined to use hand antiseptics and cologne more frequently (Figure 4). However, the participants were hesitant about vaccination for COVID-19 due to possible suspicion regarding the reliability and effectiveness of the vaccines. Distribution of the participants’ approach toward getting vaccinated with COVID-19 vaccines by demographic features and the results of the chi-squared independence test are presented in Table 4. There were statistically significant relationships between the willingness to get vaccinated and both gender and age (*p* < 0.05). As the age groups of the participants grew, their consent to vaccination increased. Moreover, occupation of the participants had a significant effect on their approach to vaccination (*p* < 0.05). On the other hand, there were no statistically significant relationship between the approach of the participants to vaccination with COVID-19 vaccines and marital status, education level, and place of residence (*p* > 0.05 for all).

## 4. Discussion

The demographic findings of this study showed that the highest number of the participants (66.4%) were detected to be at the age range between 25 and 50 years. A great majority of the participants were working (75.3%) and had a high level of education (72.2%). These groups consisted of individuals who had to work actively due to economic concerns such as financial difficulties and meeting basic needs, and therefore had the potential to come into direct contact with other people in the society. In addition, these individuals were usually exempt from the curfew imposed during certain periods of the COVID-19 pandemic. For these reasons, the risk of transmission of the disease to people in these groups was expected to be higher than the other groups. In addition, the relatively higher potential of these individuals to access the Internet and use social media actively was thought to increase their response rate to the online survey. Curfew restrictions applied to younger and older people were thought to reduce the risk of contracting the disease in these age groups.

Other risk groups exposed to restrictions in Turkey included people with a chronic illness such as chronic obstructive pulmonary disease (COPD), asthma, hypertension, and cardiovascular disease. People in this group are considered at higher risk of developing the disease with more severity and mortality rate than healthy individuals. In a study carried out by Jin et al. [10], the vast majority of COVID-19 patients (approximately 73%) was reported to have various chronic diseases, mainly hypertension and diabetes. In the present study, it was determined that 63% of the participants had one or more chronic diseases, the most frequent ones being hypertension (8.1%), allergy (7.7%), and anemia (6.6%). The fact that the immune system is suppressed in patients with chronic diseases makes them more susceptible to COVID-19, and it is therefore crucial to keep these individuals away from places with potential transmission of the disease and to implement necessary measurements more stringently. Extending scope of the restrictions to cover more chronic diseases may help prevent the spread of COVID-19 pandemic.

COVID-19 reinfections have been reported to occur in many countries, although the proportion was very low [11,12,13]. Moreover, the disease has been reported to progress more severely in individuals who contracted COVID-19 for the second time [13]. Although the reason of COVID-19 reinfections has not been clarified entirely, two different views have been put forward. According to some studies, the virus is not completely removed from the body after the first infection and causes the disease by reactivation [14]. The predominant view alleges that first infection does not produce sufficient immunity and protection in some individuals [15]. The finding of the current study that 3.9% of the participants contracted the disease twice revealed the presence of reinfections in Turkish population, albeit at a low rate. Owing to the lack of sufficient immunity in some individuals recovered from the disease, similar risk should not be ignored in case of active immunization with vaccines that may not produce an adequate response and protect from the disease. For this reason, it is very important that individuals who recovered from the disease or were vaccinated with COVID-19 vaccines should continue abiding by the measures imposed by the authorities.

Although there is no definitely effective drug in the treatment of COVID-19, hydroxychlorine sulfate used in the treatment of malaria and favipiravir used in seasonal flu are applied in the treatment of uncomplicated possible or confirmed COVID-19 cases. However, some publications reported that the incidence of cardiac arrhythmia and the mortality rate in patients using these drugs were much higher than those who did not receive the drugs for the treatment of COVID-19 [16]. Although these publications were withdrawn by the journals, they were on the agenda of the internet and television news programs. Social media channels have been reported to be the most common sources about COVID-19 despite the high potential of misinformation [17]. For this reason, people buy negative and wrong information about the drugs from these channels and act accordingly. Although there are no data regarding the acceptance of these drugs by the society, the fact that a relatively high proportion of the participants (24%) in this study did not accept treatment and turned to alternative therapies indicated that they bought negative and wrong information about the drugs. In addition, 30% of the participants stated that the drugs used in the treatment of COVID-19 cause serious side effects that may result in death. This finding suggested that doubts and hesitations about the drugs prevailed in the society.

Different results have been reported in a limited number of studies investigating the relationship between smoking and COVID-19. In a meta-analysis study conducted by Vardavas and Nikitara [18], smoking was alleged to be responsible for progression and negative consequences of COVID-19. On the contrary, the short meta-analysis of Lippi and Henry [19] revealed that there was no relationship between smoking and the severity of COVID-19. The proportion of the smoking participants (30%) in this study was similar to the officially reported general smoking rate (28%) in Turkey (TUIK) [20]. Therefore, this finding did not provide any evidence about the causal relationship between smoking and COVID-19. In order to reveal the relationship between nicotine and COVID-19, researchers must establish experimental studies. For this, databases should be determined, and analysis should focus on the role of smoking in virus contamination, the severity of and recovery from the disease. In the present study, changes in smoking attitudes of the participants after recovering from the disease were measured, and it was determined that 18% quit smoking while 27% reduced smoking. Nearly half of the participants tended to change their smoking habits, possibly worrying that the severity of COVID-19 would be triggered by smoking. As a matter of fact, 94.6% of the participants answered yes to the question “Do you pay more attention to understanding the importance of health and living a healthy life”, which supported this view.

A number of surveys have been conducted to investigate knowledge, attitudes, and behaviors related to COVID-19 in many countries following the emergence of the pandemic [4,5,6,21,22]. Most of these studies were based on collecting data from healthy individuals and showed that people generally had high levels of knowledge toward COVID-19. Likewise, the present study revealed that vast majority of the participants had sufficient knowledge about the clinical signs, transmission routes, and necessary measures to prevent transmission of the virus. Both written and visual media have played an important role in informing public rapidly and intensively about COVID-19. It has also been experienced in previous outbreaks that the society reacted rapidly to buy information in case of pandemic diseases. In fact, high rates were reported in a number of previous studies measuring KAP of people toward MERS and SARS [23,24]. Although people generally had high levels of KAP toward COVID-19, there was still confusion on some issues. For example, Azlan et al. [6] reported that only 35.7% of the participants answered no the question “The COVID-19 infection can be contracted by contact with or eating wild animals”. A similar proportion (39.5%) was obtained in the current study for the same question. While the debate about the source of the disease is still ongoing, the common opinion of the public at first was that it originated from a live market in Wuhan, China. It seems that some people still believed the accuracy of this opinion. It was also determined that the participants did not have sufficient information about the use of antibiotics in the treatment of COVID-19, since only 36.9% knew that antibiotics would not be used in the treatment. It is known that the severity and fatality of COVID-19 can increase significantly when accompanied by influenza and pneumococcal infections. For this reason, experts inform the public about having pneumococcal and flu vaccines during the pandemic. However, it was observed that this view was not largely adopted by the participants of the present study as only 26.3% answered yes to the question “Pneumococcal and flu vaccines provide a milder course of COVID-19 disease”, while 59.2% had no idea about this issue.

In this study, unlike other previous studies, KAP levels of the individuals who recovered from the disease were measured. It is expected that individuals suffering from COVID-19 should be well informed in comparison to those without the disease. However, our findings concerning the level of knowledge about COVID-19 were similar to the results of previous studies. Azlan et al. [6] reported that the knowledge scores of the participants varied significantly according to gender, age, occupation, and income level. On the other hand, Al-Hanawi et al. [4] reported that there were no statistically significant differences between knowledge scores and demographic characteristics such as marital status, education, occupation, and income level. In the present study, it was determined that demographic features such as education level, occupation, and place of residence had significant effects on the knowledge scores of the participants. The knowledge scores of those with a high level of education, living in city centers, and working as civil servants were significantly higher.

The findings of this study indicated the participants’ attitudes toward COVID-19 were not optimistic and their concerns continued at many points. Unlike other studies, only 39% of the participants believed that COVID-19 could be controlled successfully. In a study conducted in China at the beginning of the pandemic, the rate of people believing that COVID-19 could be controlled successfully was as high as 91% [9]. Likewise, high percentages (70–89%) of Chinese had optimistic attitude toward the control of SARS outbreak as well [25,26]. However, the fact that COVID-19 is still uncontrolled in many countries and continues to threat the whole world as the recent mutations are thought to increase the contagiousness of the virus might have led to decreases in optimistic attitudes of the society. The Ministry of Health spent some efforts to prevent the spread of the COVID-19 pandemic by implementing a series of measures immediately after the emergence of the first case in Turkey. These measures yielded good results, and the number of cases and deaths remained low when compared to other countries. In addition, investments made in the infrastructure of the health system in recent years compensated demands of the patients. However, only 45.2% of the participants stated that the Ministry of Health managed the pandemic well. Failure to control the COVID-19 cases despite the measures taken probably influenced the opinions of the participants. Healthcare professionals, actively involved in preventing pandemics and providing patients with the necessary medical support, have been working with great devotion. Healthcare workers are regarded as at high risk of the disease and potential carriers due to direct contact with COVID-19 patients [27]. In this study, more than half of the participants reported that they kept away from healthcare professionals because they could be potential COVID-19 carriers. One of the most striking results regarding the attitudes of the participants toward COVID-19 was that as high as 72% refrained from going to the hospital due to any illness. This situation might lead to occurrence of serious problems due to other diseases and restrict the access of people to adequate health services. In recent months, the COVID-19 virus has been reported to mutate and become more contagious in many countries, particularly in the UK. Likely depending on such news, 80% of the participants felt worried that the virus could change and become more contagious and lethal.

Significant changes were noted on the practices of the participants after recovering from COVID-19. More than 90% of the participants answered yes to the questions measuring practices related to COVID-19. However, there was no consensus among the participants on the application of COVID-19 vaccine. In studies concerning willingness of administering the COVID-19 vaccine, the vaccine acceptance rate varied by country, depending on gender, age, education level, and sociocultural characteristics. A survey conducted in the United States revealed that 67% of the participants would be willing to get vaccinated against COVID-19 if recommended by the authorities [28]. In an international survey carried out in 19 countries, it was found that the proportion of the participants accepting the use of a safe and effective COVID-19 vaccine was the highest in China with 88.6% and the lowest in Russia with 54.9% [29]. In the current study, the proportion (43.5%) of the participants willing to vaccinate was detected to be lower than those reported previously. Likewise, Salali and Uysal [30] reported that 31% of the participants in Turkey refused vaccination against COVID-19. Although the authorities are constantly informing about the safety and importance of the vaccine, the findings of the study showed that people still had doubts about COVID-19 vaccines. Beyond emphasizing the safety and effectiveness of the vaccines, it is required that new vaccine communication strategies be put in place by considering factors such as the levels of health, scientific, and general literacy of the population, and identifying locally trusted information sources.

### Limitations

The data of this study were collected online using different social media platforms (WhatsApp, Facebook, etc.) Participation to the survey was limited for people aged 51 and over, who use less social media than other age groups, and for people living in rural areas with limited access to the internet. In addition, due to the duration and urgency of the questionnaire, some factors that might affect the knowledge, attitude, and practice of the participants were not included in this study. It is thought that, despite the limitations, the findings of the study would contribute to better understanding the knowledge, attitudes, and behaviors of people who recovered from COVID-19.

## 5. Conclusions

The findings of the study indicated that the vast majority of the participants who recovered from COVID-19 had a high level of knowledge about the disease. On the other hand, most of the participants did not show optimistic attitude toward COVID-19 as they were still highly concerned about the disease. It was also noted that the behaviors of the participants following the recovery were changed positively, although there was still great instability about vaccination. In addition, it was determined that there were positive changes in the smoking habits of the participants, and most of them had one or more underlying chronic diseases. 

Future studies should focus on investigating thoroughly the reasons of public worries about COVID-19 and the measures to eliminate these worries. In addition, the most effective and reliable way of communication should be determined by conducting surveys to find out how different sources of information affect knowledge of participants and where they access the information mostly. Moreover, the reasons why the society still disapproves the vaccination, which is regarded as the most effective method of combating COVID-19, should be investigated, and more effective publications concerning the importance of vaccination should be made available on social and visual media. Finally, it is suggested that the authorities need to develop more effective and preventive strategies to find out permanent solutions in order to control the pandemic by taking into account the approaches of the public regarding COVID-19.

## Figures and Tables

**Table 1:** Demographic features of the participants (*n* = 664).

Features		Frequency (*n*)	Percentage (%)
Gender	Male	324	48.9
	Female	340	51.1
Age range (year)	15–25	124	18.7
	25–36	241	36.3
	36–50	200	30.1
	51 and more	99	14.9
Marital status	Married	425	64.0
	Never married	224	33.8
	Divorced	15	2.3
Education level	Uneducated	20	3.0
	Primary school	55	8.3
	High school	110	16.4
	Associate’s/bachelor’s degree	372	56.1
	Postgraduate	107	16.1
Residence	Province	201	30.2
	Town	425	64.1
	Village	38	5.7
Occupation	Civil servant	392	59.0
	Employee	83	12.5
	Self-employment	25	3.8
	Unemployed	164	24.7

**Table 2:** Some characteristic features of the participants associated with COVID-19.

Questions	Answers	Frequency (*n*)	Percentage (%)
Do you smoke?	Yes	199	30
	No	465	70
Has there been any change in your smoking habit after you recovered from the disease?	Likewise, I continue to smoke	109	54.8
	I reduced smoking	54	27.1
	I quit smoking	36	18.1
What type of food do you eat most?	Carbohydrate foods	164	24.7
	Meat (red and white meat, fish)	232	34.9
	Fruit/vegetables	176	26.5
	Vegan/vegetarian	6	0.9
	Balanced diet	86	13
Do you have a chronic illness?	Yes	418	63
	No	246	37
How many times did you contract COVID-19?	Once	638	96.1
	Twice	26	3.9
Where did you spend the treatment process of COVID-19?	At home	627	94.4
	Polyclinic services	34	5.1
	Intensive care unit	3	0.5
How long did your illness last?	0–10 days	384	57.8
	11–20 days	231	34.8
	21–30 days	31	4.7
	31 days and more	18	2.7
What kind of medical support did you receive during your illness?	Medication	477	71.8
	Respiratory support	3	0.5
	Medication/respiratory support	20	3
	I refused treatment	151	22.7
	Alternative treatments (herbal herbs, vitamins)	13	2

**Table 3:** The effect of demographic characteristics on the participants’ knowledge scores. (*n* = 664).

Features		Number of Participants (%)	Knowledge Score (SD)	*t/f*	*p*
Gender	Male	324 (48.9)	10.12 (2.5)	−1.30	0.192 ^a^
	Female	340 (51.1)	10.37 (2.1)		
Age range (year)	15–25	124 (18.7)	10.03 (2.6)	0.85	0.468 ^b^
	25–36	241 (36.3)	10.31 (2.3)		
	36–50	200 (30.1)	10.40 (2.3)		
	51 and more	99 (14.9)	10.08 (2.0)		
Marital status	Married	425 (64.0)	10.20 (2.3)	0.76	0.468 ^b^
	Never married	224 (33.8)	10.30 (2.4)		
	Divorced	15 (2.3)	10.93 (1.4)		
Education level	Uneducated	20 (3.0)	10.00 (2.0)	5.64	<0.001 ^b^
	Primary school	55 (8.3)	9.87 (2.4)		
	High school	110 (16.4)	9.50 (2.4)		
	Associate’s/bachelor’s degree	372 (56.1)	10.34 (2.3)		
	Postgraduate *	107 (16.1)	10.94 (2.2)		
Residence	Province	201 (30.2)	10.41 (2.3)	2.40	0.09 ^b^
	Town	425 (64.1)	10.24 (2.6)		
	Village	38 (5.7)	9.50 (2.5)		
Occupation	Civil servant	392 (59.0)	10.61 (2.4)	8.05	<0.001 ^b^
	Employee ^#^	83 (12.5)	9.50 (2.2)		
	Self-employment	25 (3.8)	9.64 (2.4)		
	Unemployed	164 (24.7)	9.85 (2.3)		

Data presented mean (x̄) and standard deviation (SD), *t/f*: t or f values. ^a^ Independent sample *t*-test. ^b^ One-way ANOVA and Tukey’s post hoc test. * Indicates statistical difference compared as high school at *p* < 0.001 level. ^#^ Indicates statistical difference compared as civil servant at *p* < 0.01 level.

**Table 4:** The relationship between approach of the participants towards vaccination from COVID-19 vaccines and demographic parameters.

Features		Yes	No	Undecided	*χ^2^*	*p* ^a^
Gender	Male *	166 (51.2%)	106 (32.7%)	52 (16.0%)	17.531	<0.001
	Female	123 (36.2%)	129 (37.9%)	88 (25.9%)		
Age range (year)	15–25	43 (34.7%)	46 (37.1%)	35 (28.2%)	17.112	0.009
	25–36	99 (41.1%)	83 (34.4%)	59 (24.5%)		
	36–50 *	97 (48.5%)	77 (38.5%)	26 (13.0%)		
	51 and more	50 (50.5%)	29 (29.3%)	20 (20.2%)		
Marital status	Married	198 (46.6%)	146 (34.4%)	81 (19.1%)	6.489	0.165
	Never married	84 (37.5%)	83 (37.1%)	57 (25.4%)		
	Divorced	7 (46.7%)	6 (40.0%)	2 (13.3%)		
Education level	Uneducated	8 (40.0%)	7 (35.0%)	5 (25.0%)	6.936	0.544
	Primary school	23 (41.8%)	23 (41.8%)	9 (16.4%)		
	High school	52 (47.3%)	34 (30.9%)	24 (21.8%)		
	Associate’s/bachelor’s degree	155 (41.7%)	130 (34.4%)	87 (23.4%)		
	Postgraduate	51 (47.7%)	41 (38.3%)	15 (14.0%)		
Residence	Province	83 (41.3%)	84 (41.8%)	34 (16.9%)	6.735	0.151
	Town	187 (44.0%)	139 (32.7%)	99 (23.3%)		
	Village	19 (50.0%)	12 (31.6%)	7 (18.4%)		
Occupation	Civil servant	189 (48.2%)	136 (34.7%)	67 (17.1%)	23.072	0.001
	Employee	30 (36.1%)	33 (39.8%)	20 (24.1%)		
	Self-employment	13 (52%)	11 (44%)	1 (4%)		
	Unemployed *	57 (34.8%)	55 (33.5%)	52 (31.7%)		

Data represented as frequency and percentage, *χ^2^:* chi-squared. ^a^ Pearson chi-squared. * Indicates statistical difference within the same features.

## Data Availability

The data presented in this study are available on reasonable request from the corresponding author.
